# Biallelic ABCG8 Variants or Biochemical Evidence of Sitosterolemia: A Case Report

**DOI:** 10.7759/cureus.115644

**Published:** 2026-09-02

**Authors:** Joseph Burns, Justin Zachariah

**Affiliations:** 1 Pediatric Cardiology, Baylor College of Medicine, Texas Children's Hospital, Houston, USA

**Keywords:** genetic disorders of lipid metabolism, pediatric cardiology, pediatric lipid disorders, preventive cardiology, sitosterolemia

## Abstract

Sitosterolemia is a rare lipid disorder characterized by xanthomas, premature atherosclerotic cardiovascular disease (ASCVD), and abnormally elevated circulating low-density lipoprotein cholesterol (LDL-c) measurements. Pathogenic variants in adenosine triphosphate (ATP)-binding cassette transporters lead to the accumulation of phytosterols. A child presented to the pediatric cardiology clinic with an incidentally noted presumed pathogenic variant and a variant of unknown significance in a sitosterolemia gene but had normal fasting lipids and no accumulation of circulating phytosterols. This case underscores the importance of phenotype-driven management and universal lipid screening, while highlighting the challenges of a gene-first testing/screening approach.

## Introduction

Disorders of lipid metabolism are common in pediatric populations, affecting approximately one in five children aged 6 to 17 [1,2]. Monogenic dyslipidemias are also relatively common Mendelian genetic disorders, characterized by severe lipid elevations [3]. Among these, familial hypercholesterolemia (FH), characterized by markedly elevated low-density lipoprotein, is the most prevalent, affecting 1 in 250 to 500 live births [4,5]. While severely elevated measured low-density lipoprotein cholesterol (LDL-c) is usually attributed to FH, there are other explanatory conditions, including sitosterolemia.

Sitosterolemia is caused by elevated circulating levels of plant sterols, or phytosterols, due to increased absorption of plant sterols and diminished excretion [6,7]. Approximately one in 200,000 individuals are affected by sitosterolemia [7]. Affected patients typically can present with abnormal LDL-c, often FH level but sometimes lower, xanthomas, and/or premature atherosclerotic cardiovascular disease (ASCVD) [7]. These examination and laboratory findings mimic FH and commonly lead to misdiagnosis, as plant sterols are not assessed on standard lipid assays. Thus, sitosterolemia must be considered in the differential diagnosis for such patients, especially as affected persons may be unresponsive to statin therapy, which can be misinterpreted as noncompliance.

*ABCG8 *and *ABCG5 *are causally implicated genes encoding adenosine triphosphate (ATP)-binding cassette transporters in enterocytes and hepatocytes that decrease absorption and promote the excretion of phytosterols [8]. Biallelic mutations in these genes are known to cause sitosterolemia [8,9]. Sitosterolemia is typically described as autosomal recessive, caused by biallelic pathogenic variants in *ABCG8* or *ABCG5*. Sitosterolemia is typically described as autosomal recessive, caused by biallelic pathogenic variants in *ABCG8 *or *ABCG5* [10]. This compound heterozygosity generally yields the most severe phenotype, though heterozygous patients with a single pathogenic variant also exhibit elevated sitosterol levels in *ABCG5 *mutations [10].

This case describes a child presenting for preventive cardiology care due to incidentally identified *ABCG8 *variants with incompletely characterized pathogenicity without the biochemical phenotype of sitosterolemia, highlighting the importance of phenotype-driven management and universal lipid screening over a gene-first approach.

## Case presentation

The patient is a seven-year-old girl who was born at term. She has a history of recurrent, severe respiratory infections with chronic cough requiring multiple rounds of antibiotics and systemic steroids. She was then seen by pulmonology and underwent a chest computed tomography, which revealed subsegmental atelectasis without bronchiectasis. She was additionally evaluated by gastroenterology and otolaryngology, with normal endoscopy, but tracheomalacia and left bronchomalacia were noted on direct laryngoscopy and bronchoscopy. A sleep study revealed severe obstructive sleep apnea (apnea-hypopnea index 10.2/hour), prompting tonsillectomy and adenoidectomy. She was also found to have low immunoglobulins, prompting consultation with allergy and immunology. Apart from her history of respiratory infection, she had no other symptoms, including chest pain, palpitations, syncope, or skin symptoms.

Further questioning revealed no significant family history of premature ASCVD nor a lipid disorder. Both parents were asymptomatic, though the maternal variant was suggested to be disease-associated on clinical laboratory reporting, as above. However, neither variant is classified as pathologic in the ClinVar database [11,12].

At presentation, her medications were loratadine, a multivitamin not containing plant sterols, and a probiotic supplement. Physical examination revealed a BMI of 27.25 kg/m^2^, no murmurs, rubs, or gallops on auscultation, and no evidence of xanthomas or xanthelasmas. Her diet was described as typical, with no significant plant avoidance.

The persistence of the respiratory symptoms prompted genetic testing for primary ciliary dyskinesia and cystic fibrosis. Neither of these conditions was genetically confirmed, but testing revealed incidentally identified ABCG8 variants of incompletely characterized pathogenic significance. Parental testing ensued, revealing that the maternal variant (c.55G>C (p.Asp19His)) was of increased pathogenicity risk and the heterozygous paternally inherited variant was of unknown significance (c1505C>T(p.Pro502Leu)) per the clinical laboratory reports. Given that biallelic mutations in ABCG8 are associated with sitosterolemia, she was referred to the preventive cardiology service. Her initial laboratory testing is summarized in** **Table 1.

**Table 1:** Initial and follow-up fasting lipid panels

Parameter	Reference range	First results	Repeat results (3 months later)
Total cholesterol	<170 mg/dL	136 mg/dL	122 mg/dL
High-density lipoprotein cholesterol	>55 mg/dL	49 mg/dL	44 mg/dL
Triglycerides	<75 mg/dL	88 mg/dL	63 mg/dL
Low-density lipoprotein cholesterol	<110 mg/dL	70 mg/dL	64 mg/dL
Hemoglobin A1c	<5.7%	5.2%	5.1%
Lipoprotein (a)	<75 mmol/L	23 nmol/L	13 nmol/L

This was repeated to ensure normalcy (Table 1), with the only abnormality being her relatively low high-density lipoprotein cholesterol for age. Though reassured by these results, further testing to evaluate for sitosterolemia was obtained. This assessment is summarized in Table 2.

**Table 2:** Advanced testing of plasma sterols DiHydro T-MAS: dihydrotestis meiosis-activating sterol

Parameter	Result (mg/L)	Interpretation
7-dehydrocholesterol	0.4	Normal (<2.0 mg/L)
8-dehydrocholesterol	0.2	Normal (<0.3 mg/L)
8(9)-cholesterol	<1.0	Normal (<5.0 mg/L)
Campesterol	2.4	Normal (<8.0 mg/L)
Cholestanol	1.4	Normal (<6.0 mg/L)
Demosterol	0.9	Normal (<2.5 mg/L)
DiHydro T-MAS	0.1	Normal (<0.3 mg/L)
Lathosterol	1.7	Normal (<15.0 mg/L)
Squalene	0.2	Normal (<1.0 mg/L)
Stigmasterol	0.1	Normal (<0.5 mg/L)

This testing was not suggestive of sitosterolemia, though it did not test for beta-sitosterol, another plant sterol that may accumulate in sitosterolemia; this may be required in future testing if her LDL-c is found to be elevated for age.

Based on these results, the patient and family were counseled that there is currently no evidence of sitosterolemia. Dietary counseling to promote healthy lipid levels was provided to reduce future ASCVD risk, a concern for all patients. She was discharged from preventive cardiology care but advised to repeat the fasting lipid panel every one to two years with her primary care physician and return should she develop abnormal lipid disorders, including abnormal LDL-c for age, which may prompt repeat assessment of sterols. Consent was obtained from the family to proceed with this case report.

## Discussion

In this patient, the absence of clinical or laboratory findings of sitosterolemia may be attributable to incomplete penetrance of her potentially pathogenic ABCG8 mutation or to the possible clinical insignificance of the variant of unknown significance, particularly as neither variant is classified as pathologic in ClinVar, which also may account for her normal plasma sterol testing. However, because beta-sitosterol was not measured directly, her genotype-phenotype relationship is not fully characterized. Alternative hypotheses include residual activity from hypomorphic alleles, low dietary consumption of plant sterols, or compensatory mechanisms. Thus, she is best characterized as carrying biallelic ABCG8 mutations of unlikely pathogenic significance, given the absence of sitosterolemia on biochemical testing, with the limitation of the lack of testing for beta-sitosterol, which is hypothesized to be normal given normal LDL-c. However, prediction tools may demonstrate a pathologic functional impact in further testing.

In patients with sitosterolemia, treatment focuses on dietary elimination of plant sterols, which are markedly elevated on laboratory testing [7]. In patients in whom this is inadequate to reduce sterol levels, ezetimibe is considered a first-line therapy with bile acid sequestrants as a possible adjunct [7]. The use of ezetimibe is particularly direct, as inhibition of Niemann-Pick C1-like 1 protein at the enterocyte brush border reduces phytosterol absorption, thereby preventing the accumulation of phytosterols in sitosterolemia. Likewise, bile acid sequestrants can alter the enterohepatic circulation, thereby preventing phytosterol reabsorption. Generally, sitosterolemia is poorly described in children, and this case may contribute to the existing body of knowledge in this disorder [13]. Similarly, given the relatively small number of affected patients, this supports the study of variants known to be associated with phenotypic sitosterolemia [14]. 

For this patient, referral based on incidentally identified variants without biochemical evidence of sitosterolemia was likely premature. In patients with sitosterolemia, the typical screening approach recommended by the National Heart, Lung, and Blood Institute is likely to identify affected patients. This recommendation promotes universal screening with a nonfasting lipid profile, followed by fasting testing if the nonfasting result is abnormal [15,16]. Currently, the standard of care for children with FH-level LDL-c, as seen in sitosterolemia, is medical management without genetic testing. When the clinical trajectory is unexpected, such as with xanthomas in youth with heterozygous FH and LDL-c levels or a lack of response to statin therapy, genetic testing may be considered to identify rare lipid disorders, including sitosterolemia. Specialized testing for circulating phytosterols may also be confirmatory.

## Conclusions

This case underscores the importance of careful, thoughtful interpretation of genetic information and biochemical confirmation before assigning a diagnosis of a rare lipid disorder. Primary genetic screening would be a complex tool; in this patient's case, genotype does not always translate into a clinical phenotype in genetic lipid disorders, and the lack of testing for beta-sitosterol limits certainty. We hypothesize that her biallelic variants likely do not harbor pathologic consequences, but should her LDL-c become abnormal in the future, we will surely re-engage and pursue further testing. Also complicated are findings that genotype-positive lipid disorders with lower distributions of the phenotype may still be at elevated ASCVD risk, giving pause to completely discharging patients like this, as in a “gene-first” approach, in favor of lifelong surveillance.
